# Forecasting effects of “fast-tracks” for surgery in the Swedish national guidelines for distal radius fractures

**DOI:** 10.1371/journal.pone.0260296

**Published:** 2022-02-10

**Authors:** Viktor Schmidt, Cecilia Mellstrand Navarro, Marcus Ottosson, Magnus Tägil, Albert Christersson, Markus Engquist, Arkan Sayed-Noor, Sebastian Mukka, Mats Wadsten

**Affiliations:** 1 Department of Surgical and Perioperative Sciences (Orthopedics), Umeå University, Umeå, Sweden; 2 Department of Clinical Science and Education, Södersjukhuset, Karolinska Institutet, Stockholm, Sweden; 3 Department of Hand Surgery, Södersjukhuset, Stockholm, Sweden; 4 Department of Clinical Sciences Lund, Orthopedics, Lund University, Lund, Sweden; 5 Department of Orthopedics, Institution of Surgical Sciences, Uppsala University, Uppsala, Sweden; 6 Department of Orthopedics, Ryhov Hospital, Jönköping, Sweden; Assiut University Faculty of Medicine, EGYPT

## Abstract

**Background and purpose:**

National guidelines for treatment of distal radius fractures (DRFs) were presented in Sweden in 2021. In the guidelines, a fast-track is recommended for 4 subgroups of highly unstable DRFs. Regardless of the results of the closed reduction these are recommended for surgery within 1 week of injury. This study aims to evaluate the potential consequences of the newly presented national guidelines on incidence of surgical interventions.

**Patients and methods:**

In all, 1,609 patients (1,635 DRFs) with primary radiographs after a DRF between 2014 and 2017 at two Swedish hospitals were included in a retrospective cohort study. An estimation was made of the percentage of patients in the historical pre-guidelines cohort, that would have been recommended early primary surgery according to the new national guidelines compared to treatment implemented without the support of these guidelines.

**Results:**

On a strict radiological basis, 32% (516 out of 1635) of DRFs were classified into one of the 4 defined subgroups. At 9–13 days follow-up, cast treatment was converted into delayed primary surgery in 201 cases. Out of these, 56% (112 out of 201) fulfilled the fast-track criteria and would with the new guidelines have been subject to early primary surgery.

**Interpretation:**

The fast-track regimen in the new guidelines, has a high likelihood of identifying the unstable fractures benefitting from early primary surgery. If the proposed Swedish national guidelines for DRF treatment are implemented, a greater proportion of fractures would be treated with early primary surgery, and a delayed surgery avoided in the majority of cases. The potential benefits in relation to possible costs when using the fast-track criteria in every day practice are still unknown.

## Introduction

Distal radius fracture (DRF) is the most common fracture in adults [1, 2] and the incidence is increasing worldwide [2, 3]. Thus, DRFs represent a large part of emergency department visits [3].

Fracture treatment is decided based on radiological features and patient-related factors. What kind of surgery to recommend is under debate, as is surgical timing [4–9]. During the 21^st^ century, preferences towards surgical intervention have increased [10–13]. In displaced fractures surgical treatment is more likely to restore anatomical alignment than nonsurgical treatment [14, 15], which could be important in obtaining good clinical outcomes [15]. Moreover, there are indications that early primary surgery provides better outcomes for hand function than delayed primary surgery [6, 14].

Due to conflicting definitions for acceptable radiological alignment [16–26], there are differences both between [21–26] and within countries [12, 13, 27–29] regarding treatment recommendations for DRFs.

To avoid unwarranted treatment variations several national guidelines have been published in recent years [21–26]. In April 2021, Swedish national guidelines [30] were presented by a multi-professional group working on behalf of the Swedish Association of Local Authorities and Regions (SALAR, SKR).

The Swedish national guidelines have identified 4 subgroups of radiological features of fractures that, due to inherent instability, are considered to have a minimal chance of successful nonoperative treatment, defined as healing in an acceptable position. To minimize treatment-delay the Swedish national guidelines recommend that patients with these fractures undergo surgery within 1 week of injury without further radiological follow-up visits, regardless of radiological alignment after closed reduction. Treatment recommendations of this type have not been proposed in other national guidelines [21–26].

This study aims to evaluate whether and to what extent the newly introduced fast-track regimen will affect the proportions of fractures treated with early and delayed primary surgery.

## Patients and methods

### Study design and settings

This retrospective cohort study included 1,609 consecutive patients treated for DRF (1,635 DRFs) at Sundsvall Hospital and Umeå University Hospital between January 2014 and January 2018. The Sundsvall Hospital is a second referral hospital with a catchment area of 150,000 inhabitants. Umeå University Hospital is a tertiary referral hospital with a catchment area of approximately 160,000 inhabitants. We designed and conducted the study after the development of new national guidelines to evaluate the potential effects on fracture care of a fast-track treatment regimen suggested in the new guidelines.

### Participants and data collection

**Fig 1:**
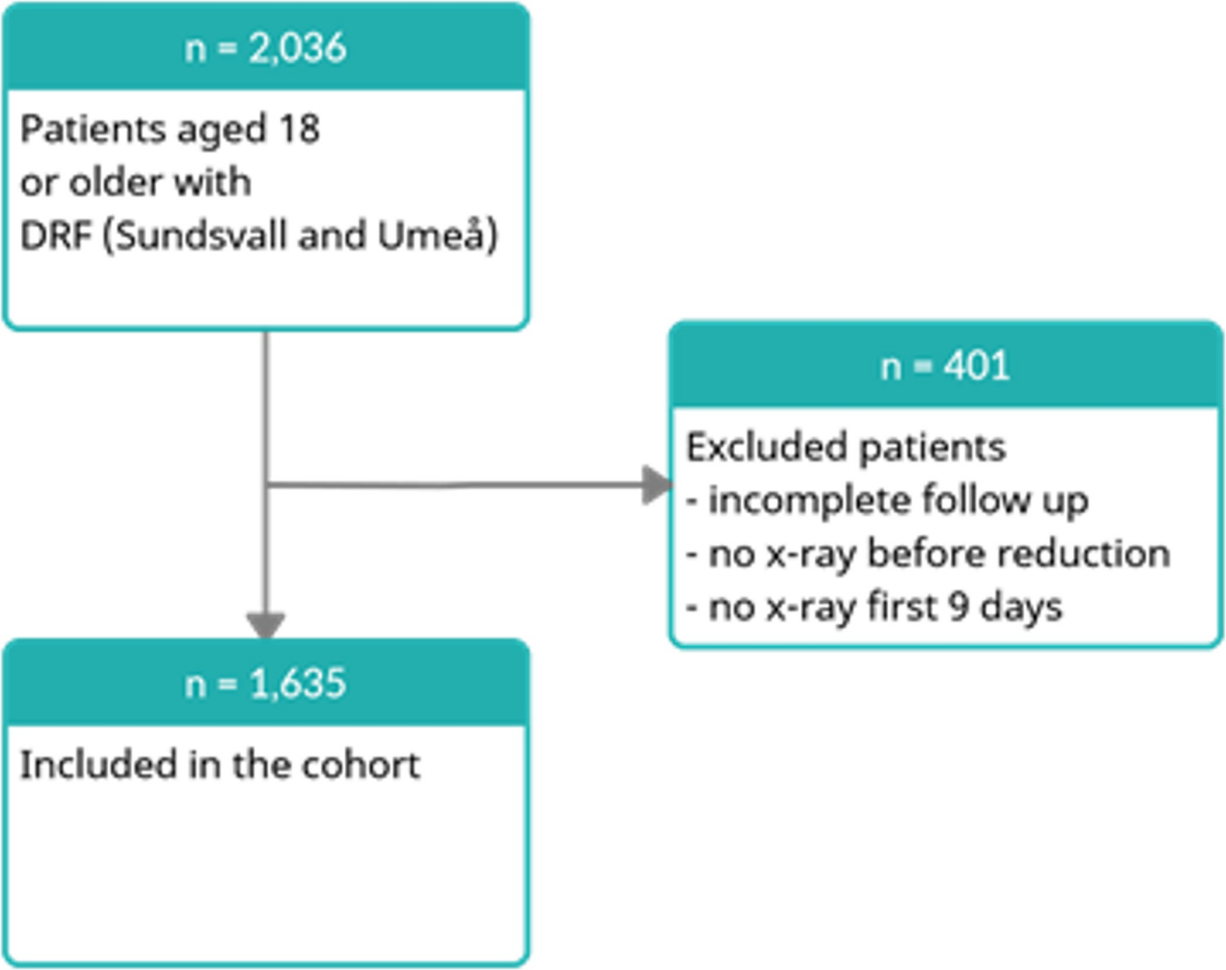
Flowchart of patient inclusion and exclusion in a retrospective cohort study of 1,635 distal radius fractures (DRF) in two hospitals in Sweden in 2014–2017.

The study population (all patients > 18 years of age with a DRF) was identified through an electronic database search for the ICD-10 code [31] for a DRF (ICD S52.5 and S52.6) at Sundsvall Hospital and Umeå University Hospital. The data were collected through analysis of radiographs and a review of the medical records. The ethics committee waived the requirement for informed consent. We excluded patients without a primary radiograph before reduction or within 9 days of injury (Fig 1).

Data was collected from the medical records. Operative or nonoperative treatment was noted, and if operated, whether surgery was early or delayed. Early primary surgery was defined as a surgery decision based on radiographs from the first health care visit only. Delayed primary surgery was defined as surgery decided after the outpatient clinic visit at 9-13-days. Additional clinical data such as age, sex, suspected osteoporosis, diagnosed dementia (yes/no), domestic care services (yes/no), institutional living (yes/no) and fracture characteristics were recorded. Data on cognitive impairment, domestic care services and institutional living were collected and assessed through a patient record review until the date of injury. For all patients, treatment was according to surgeons preferred method and before the Swedish national guidelines were established. In general, this meant that treatment after surgery was: 5 weeks immobilization after pinning or external-fixation and 2 weeks after volar plating. After cast/external fixator removal, therapy was initiated either as a home exercise program or together with an occupational therapist.

### Summary of the Swedish national guidelines

The Swedish National Guidelines for treatment of distal radius fractures were developed in 2019–2021 by an interprofessional expert group consisting of orthopedic surgeons, hand surgeons, occupational therapists, physiotherapists, plaster technicians and patient representatives in order to provide health care staff support in their decision-making regarding patients with wrist fractures. The guidelines were synthesized based on existing local and international treatment recommendations, published scientific literature and the clinical experience in the expert group. The guidelines address the most common issues related to treatment and rehabilitation of distal radius fractures in adults. The complete version is available in Swedish online [30]. A summary of the guidelines in English is provided in Appendix 1.

Most treatment recommendations use patient age as the principal criterion for decision making. In contrast, the Swedish national guidelines for DRFs advise that the treatment choice is based on the patients’ functional demands, rather than age. The guidelines suggest three defined functional levels: High, intermediate or low functional demands. Criteria for each functional level are presented in Table 1 [30].

**Table 1:** Functional demands and treatment thresholds as presented by the Swedish national guidelines for DRFs [30].

Functional demands	Interpretation	Acceptable alignment
High	Need to use the hand in heavy labor or activities in work, free time or daily activities.	• Dorsal angulation < 10° • Volar angulation < 15° • Radial inclination > 15° • Ulnar variance < 2 mm shortening • Intra-articular step < 2 mm • Volar cortex continuity • Coronal shift < 2 mm • Congruent DRU-joint
Intermediate	Need to use the hand in activities of daily living (ADL) independently, but without the need to load the wrist heavily in a physical labor or spare-time activity.	• Dorsal angulation < 20° • Volar angulation < 15° • Radial inclination > 10° • Ulnar variance < 3 mm shortening • Intra-articular step < 2 mm • Volar cortex continuity • Congruent DRU-joint
Low	Permanent incapability to independently perform activities of daily living (ADLs).	No skin, nerve, or circulatory compromise

In the Swedish national guidelines for DRF treatment all fractures are judged based on radiographic findings. Nonsurgical treatment and cast immobilization is recommended for undisplaced fractures. In displaced fractures, closed reduction and casting are recommended. If closed reduction fails to restore anatomy within the stated limits, early primary surgery, defined as surgery within 1 week of injury, is recommended. For most patients undergoing nonoperative treatment, a radiographic control at 9–13 days is recommended and if the fracture alignment is poor, delayed primary surgery is advised. Surgery could be considered even for minor redisplacement at the 9-13-day control if displacement has occurred, although within the threshold.

**Fig 2:**
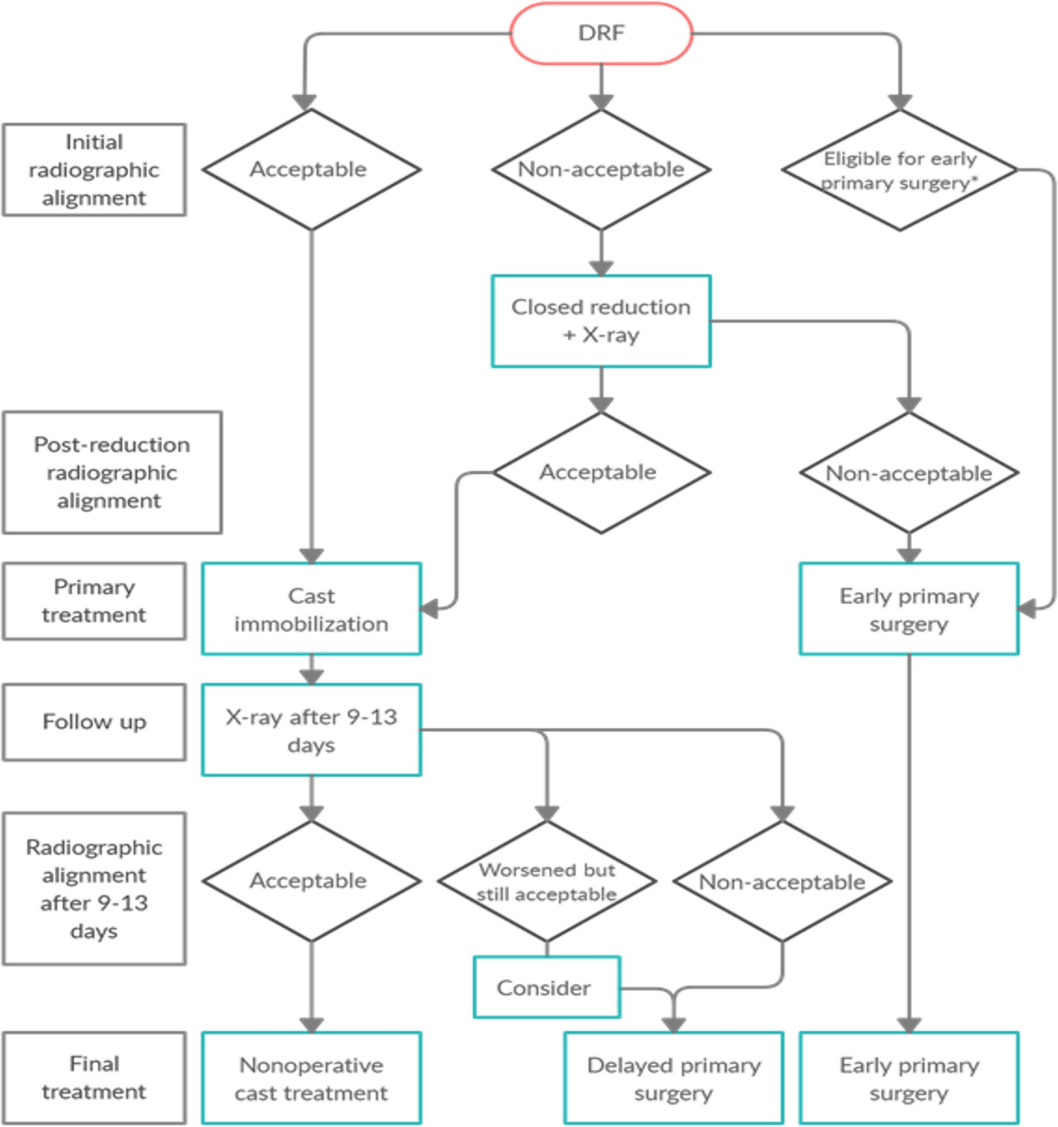
Flow chart for treatment suggested in the Swedish national treatment guidelines for distal radius fractures (DRF). *According to the 4 defined subgroups (fast-tracks) analyzed in the present study.

**Fig 3:**
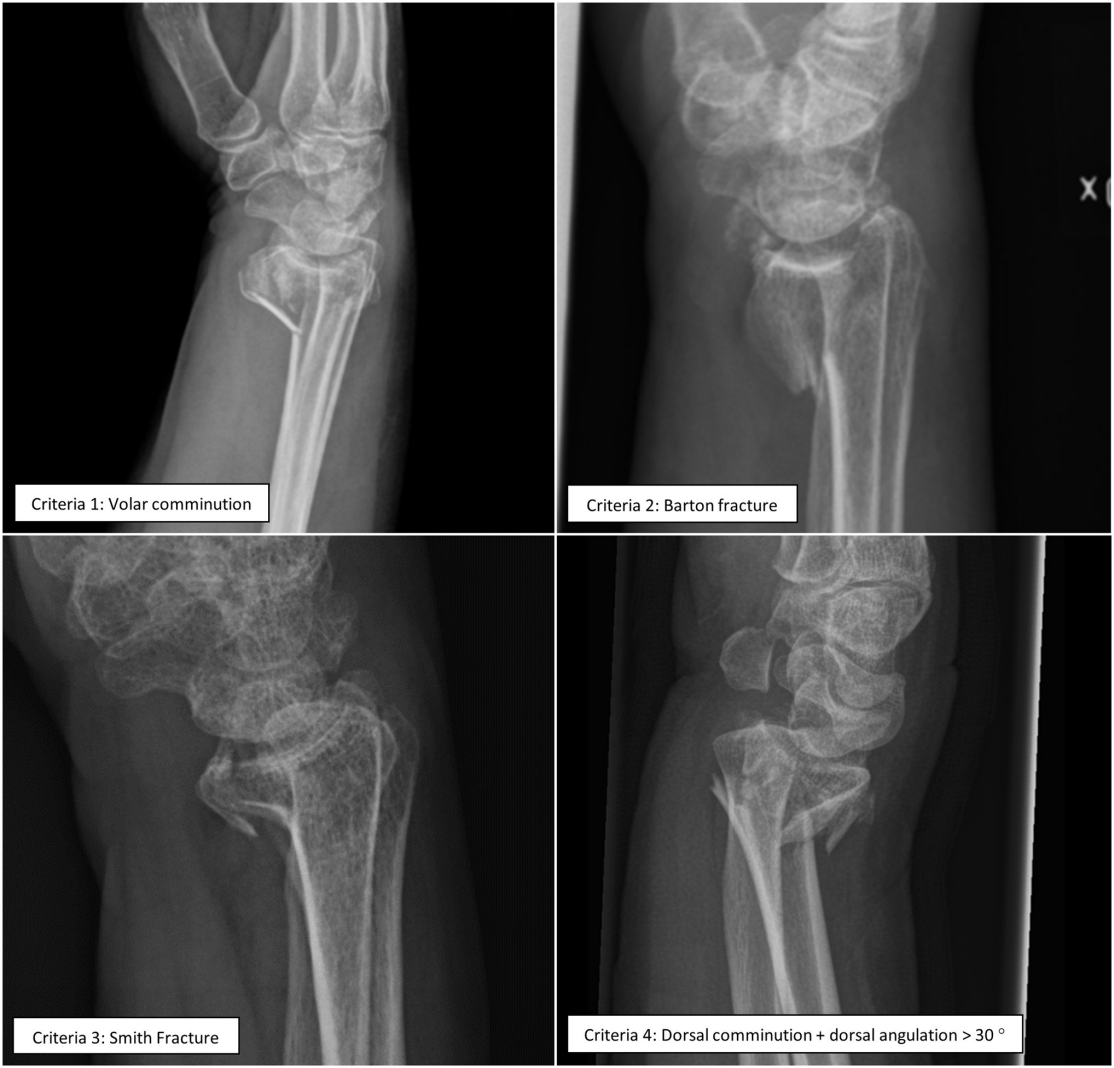
Examples of fractures for each of the defined subgroups suggested in the Swedish national treatment guidelines for distal radius fractures.

In addition to the part of the guidelines using radiographic displacement as a proxy for stability, a second part was suggested. In this, the fracture type was used to predict loss of position between the time of fracture and the radiographic follow up. The present study centers on the 4 subgroups of DRF patients identified in the Swedish national guidelines to be suitable for early primary surgery, a fast-track, regardless of the closed reduction results (Fig 2). The Swedish national guidelines for DRF propose that, in patients with high or intermediate functional demands (Table 1), the following fracture subgroups should be considered for early primary surgery, even if acceptable alignment has been achieved after closed reduction and casting. Clinical examples of each subgroup are depicted in Fig 3.

1. Volar comminution, with comminution defined as a free-floating piece of cortex > 3 mm [32].
2. Barton fracture (volar or dorsal) with displacement [33].
3. Smith fracture [33].
4. Simultaneous presence of dorsal comminution (a free-floating piece of cortex > 3 mm [32]), severe initial displacement (dorsal angulation > 30°, radial inclination < 10°, or ulnar variance > 3 mm), and clinical suspicion of physiological osteopenia/osteoporosis*.

*Sufficient data on clinical signs of physiological osteoporosis were not present for all individuals. Therefore, age > 50 years was used as a proxy for osteopenia/osteoporosis in this study [34].

### Radiographic analysis

**Fig 4:**
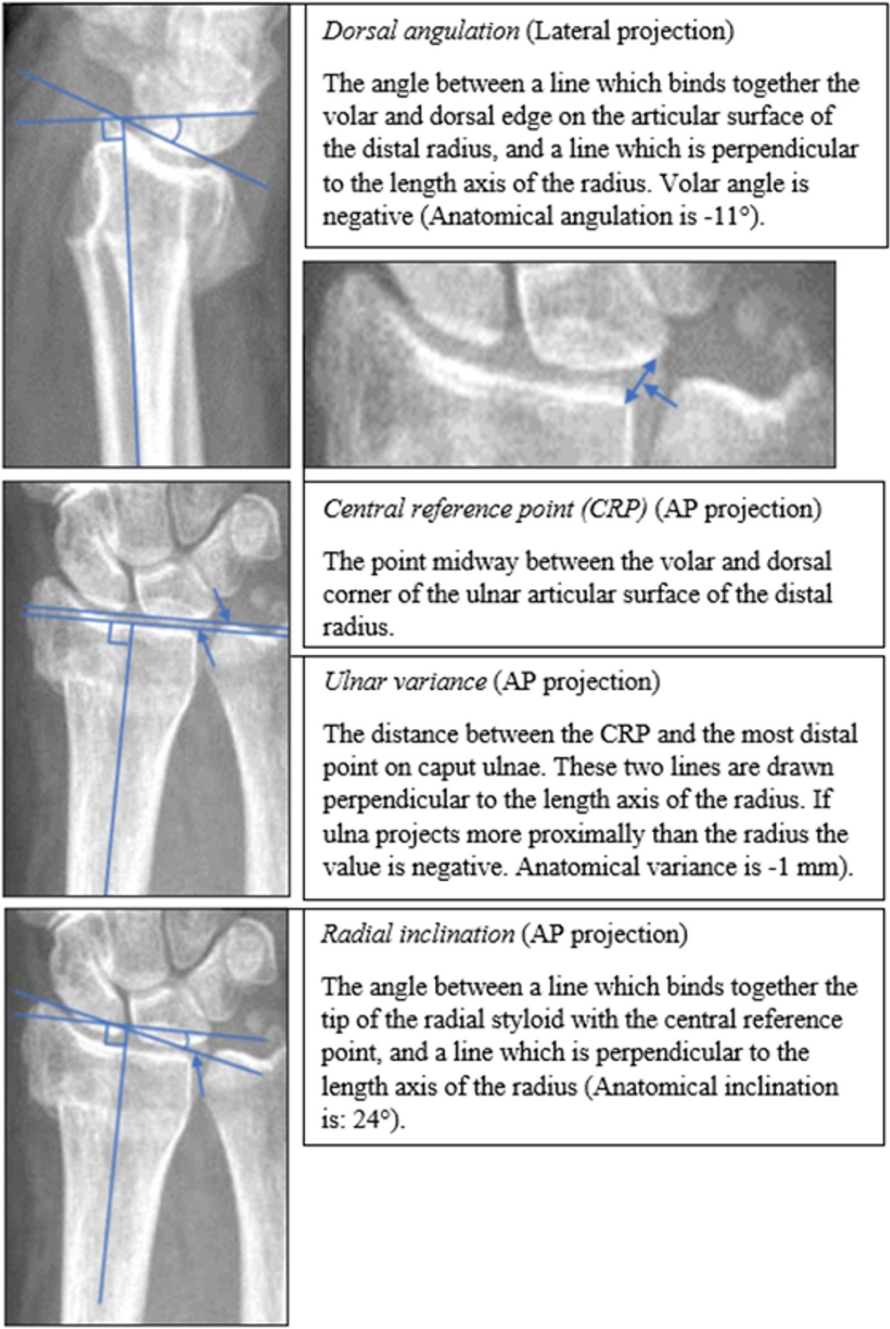
How radiological measurements were made in a retrospective cohort study of 1,635 DRFs in two hospitals in Sweden in 2014–2017.

Three of the present authors (MW, VS, MO) performed the radiological measurements (Table 2). All measurements were performed on conventional wrist x-ray images (anteroposterior and lateral). Radial inclination, ulnar variance, dorsal tilt, intra-articular fracture and presence of comminution according to the guidelines’ definition (dorsal/volar) were assessed on the primary radiograph (Fig 4).

**Table 2:** Interrater reliability was measured between three raters with intraclass correlation coefficient in a retrospective cohort study of 1,635 DRFs in two hospitals in Sweden from 2014–2017. Measures < 0.50 are poor, between 0.50 and 0.75 moderate, between 0.75 and 0.90 good and > 0.90 excellent.

Intraclass correlation		
	Average measures	Interpretation
**Dorsal angulation**	0.998	Excellent
**Radial inclination**	0.970	Excellent
**Ulnar variance**	0.931	Excellent
**Intra-articular step**	0.972	Excellent

All fractures that met the criteria for any of the 4 subgroups, as proposed for early primary surgery in the national guidelines (Fig 3) were identified.

### Outcomes

The proportion of patients fulfilling any of the radiological criteria for early primary surgical treatment according to the defined subgroups in the Swedish national guidelines for DRF treatment, served as primary outcome. Proportions were presented for each fracture subgroup.

Secondary outcome was the discrepancy between given treatment and treatment recommended by the guidelines (Fig 2) presented as the proportion of patients who would have been treated with early primary surgery instead of delayed primary surgery. Proportions were presented for each of the 4 criteria separately and for the total groups recommended for early primary surgery.

## Statistics

Numerical data were expressed as mean and standard deviation (SD) and median and interquartile range (IQR). Categorical data were presented as numbers and proportions and a corresponding 95% confidence interval (CI). A multivariate multinomial logistic regression model was used to analyze the 4 subgroups for surgery (hereafter called “fast-tracks”) (Table 3). Parameters that were significant in univariate analysis and available confounders were entered in the model. The outcome was final treatment (nonoperative, early primary surgery or delayed primary surgery) and the variables were age (continuous data), sex (nominal data), intra-articularity (nominal data) and fast-tracks (categorical data). In univariate analysis age was analyzed with ANOVA while sex, intra-articularity and fast-tracks were analyzed with chi-squared test. A p-value of < 0.05 was considered significant.

**Table 3:** Multivariate analysis with multinomial linear regression for treatment (nonoperative, early or delayed primary surgery) as the dependent variable in a retrospective cohort study of 1,635 DRFs in two hospitals in Sweden in 2014–2017.

				95% Confidence interval for OR^a^	
Treatment^b^		P-value	OR	Lower bound	Upper bound
Early primary surgery	Age	.000	.968	.959	.978
	FT^c^ 4	.000	13.496	9.188	19.825
	FT^c^ 3	.000	21.711	5.830	80.848
	FT^c^ 2	.000	81.898	17.538	382.451
	FT^c^ 1	.000	21.207	13.847	32.481
	FT^c^ 0	Reference	Reference	.	.
	IA^d^ 0	.292	.848	.623	1.153
	IA^d^ 1	Reference	Reference	.	.
	Male	.118	.733	.496	1.083
	Female	Reference	Reference	.	.
Delayed primary surgery	Age	.000	.979	.968	.989
	FT^c^ 4	.000	6.531	4.363	9.776
	FT^c^ 3	.001	12.050	2.901	50.049
	FT^c^ 2	.001	24.086	3.912	148.314
	FT^c^ 1	.000	9.422	5.863	15.143
	FT^c^ 0	Reference	Reference	.	.
	IA^b^ 0	.044	1.400	1.009	1.943
	IA^b^ 1	Reference	Reference	.	.
	Male	.001	.457	.284	.736
	Female	Reference	Reference	.	.

a. OR = Odds ratio.

b. The reference category is Nonoperative.

c. FT = Fast-track, where 1 is Volar, 2 is Barton, 3 is Smith, 4 is Combination and 0 is none.

d. IA = Intra-articular, where 0 is no and 1 is yes.

Intraclass correlation coefficient was used to measure interrater reliability for the radiological assessments.

Statistical analysis was performed using IBM SPSS Statistics (V26).

## Results

### Patients and descriptive data

1,609 patients with DRF (1,635 DRFs) were included. 79% (CI 76–80%) were women and the mean age was 61 years (SD 17) and median 63 years (IQR 52–72). 5% (CI 4–7%) of the patients over 65 had domestic care services.

18% (CI 16–20%) of the fractures were treated with early primary surgery (median 3, IQR 1–5 days until surgery) and 15% (CI 13–17%) of initially nonoperatively treated fractures were treated with delayed primary surgery (median 13, IQR 12–15 days until surgery). In total 30% (CI 28–33%) of the fractures were treated surgically.

### Outcomes

#### Primary outcome

32% (CI 29–34%) of all the DRFs fulfilled radiological criteria for any 4 fast-track fracture types.

**Fig 5:**
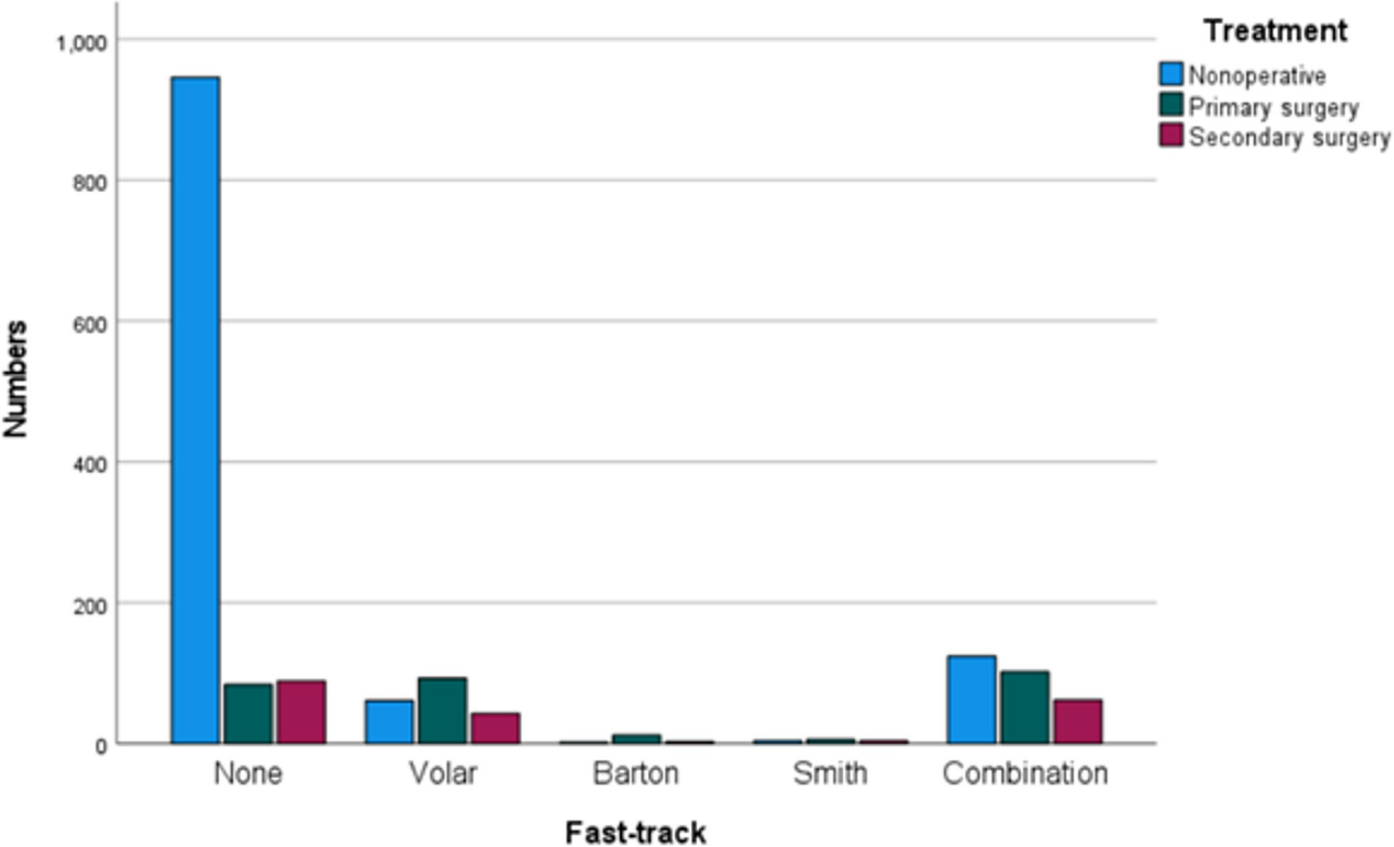
Bar chart depicting differences in treatment depending on fracture type in a retrospective cohort study of 1,635 DRFs in two hospitals in Sweden in 2014–2017. None: Fracture did not fulfill the criteria for any of the categories listed below. Volar: Volar comminution, defined as a free-floating piece of cortex > 3 mm. Barton: Intra-articular volar or dorsal fracture with displacement of the articular surface. Smith: Volar displacement of the distal fragment. Combination: Simultaneous presence of dorsal comminution, severe initial displacement (dorsal angulation > 30° or radial inclination < 10° or ulnar variance > 3 mm) and suspected physiological osteopenia/osteoporosis.

12% (CI 11–14%) displayed volar comminution, 1% (CI 0.6–1.7%) had a displaced Barton-fracture, 0.9% (CI 0.5–1.4%) a Smith-fracture and 18% (CI 16–20%) fulfilled the combination-criteria of the simultaneous presence of dorsal comminution, severe initial displacement and suspected physiological osteopenia/osteoporosis (Fig 5).

63% (CI 59–67%) of the fractures fulfilling criteria for the defined fast-tracks were surgically treated (Table 4).

**Table 4:** Presentation of fracture treatment depending on fracture type in a retrospective cohort study of 1,635 DRFs in two hospitals in Sweden in 2014–2017.

		Nonoperative	Early primary surgery	Delayed primary surgery	Total
**None** ^**a**^		946 (84.5%)	84 (7.5%)	89 (8.0%)	1119
**Fast-track**	**All** ^**b**^	191 (37.0%)	213 (41.3%)	112 (21.7%)	516
	**Volar** ^**c**^	61 (31.0%)	93 (47.2%)	43 (21.8%)	197
	**Barton** ^**d**^	2 (11.8%)	12 (70.6%)	3 (17.6%)	17
	**Smith** ^**e**^	4 (28.6%)	6 (42.9%)	4 (28.6%)	14
	**Combination** ^**f**^	124 (43.1%)	102 (35.4%)	62 (21.5%)	288
**Total**		1137 (69.5%)	297 (18.2%)	201 (12.3%)	1635

a. Fracture did not fulfill any of the fast-tracks.

b. Fracture fulfilled any one of the fast-tracks.

c. Volar comminution, defined as a free-floating piece of cortex > 3 mm.

d. Intra-articular volar or dorsal fracture with displacement of the articular surface.

e. Volar displacement of the distal fragment.

f. Simultaneous presence of dorsal comminution, severe initial displacement (dorsal angulation > 30° or radial inclination < 10° or ulnar variance > 3 mm) and suspected physiological osteopenia/osteoporosis.

Of the fractures fulfilling the criteria for fast-tracks, only 41% (CI 37–46%) were treated with early primary surgery, and 59% (CI 54–63%) were initially treated with a cast. Of those fulfilling the criteria for fast-tracks that were initially treated with a cast, 37% (CI 32–43%) were treated with delayed primary surgery. In total 63% (CI 59–67%) of fractures fulfilling fast-track criteria were treated surgically.

#### Secondary outcome

Of all fractures treated with delayed primary surgery (regardless of fracture type), 56% (CI 49–63%) fulfilled the fast-track criteria and would have been recommended early primary surgery according to the guidelines (Table 5) (Umeå University hospital 73%, Sundsvall Hospital 48%).

**Table 5:** Presentation of how treatment could change with the new guidelines and implemented fast-track based on a retrospective cohort study of 1,635 distal radius fractures (DRF) in two hospitals in Sweden in 2014–2017.

Treatment	Without guidelines	Change (n, %)	With guidelines
Early primary surgery	297 fractures	+112 (+38%)	409 fractures
Delayed primary surgery	201 fractures	‒112 (‒56%)	89 fractures

#### Treatment

68% (CI 66–71%) did not fulfill the criteria for any of the 4 fast-tracks. Of those, 8% (CI 6–9%) were treated with early primary surgery and, of the fractures initially treated nonoperatively, 9% (CI 7–10%) were treated with delayed primary surgery after radiological control. 15% (CI 13–18%) of the fractures that did not fulfill the criteria for any of the 4 fast-tracks were treated surgically.

A positive correlation between fast-track radiological criteria and surgical treatment was found (Table 3), indicating that DRFs that meet the criteria for fast-track were much more likely to be treated with primary surgery. Findings were significant in univariate and multivariate analysis adjusting for age, sex and intra-articular fracture (p<0.001) (Table 3).

Interrater reliability of radiological assessments was excellent (Table 5).

## Discussion

Nonoperative treatment in a cast is less stable for DRFs than surgical fixation, with malunion a well-known complication [15]. Displacement during nonsurgical treatment is common and delayed primary surgery is often necessary. To minimize unnecessary treatment delays the Swedish national guidelines for treating DRFs introduced fast-tracks for 4 defined subgroups of highly unstable fracture types. According to our results, almost one of three DRFs fulfilled the radiological criteria for any of the fast-tracks and would potentially be recommended for early primary surgery. At the same time, the total number of patients subject to treatment with delayed primary surgery would be avoided in a majority of cases in a setting where the new guidelines are fully implemented. We believe that healthcare systems would benefit from limiting the number of patients planned for radiographic follow-up and delayed surgery.

The 4 fast-tracks were defined based on inherent fracture instability. Volar comminution is a highly unstable fracture characteristic [35]. This particular fracture type was also fairly common in the present study (12%). Barton and Smith fractures are known for their complexity and instability; however, they were quite rare (1.0% and 0.9%, respectively). The combination fast-track (18%) described in the Swedish national guidelines was created because there is evidence of instability in dorsally comminuted fractures and older patients [36, 37]. A combination of these two factors and an initial severe displacement create a highly unstable fracture pattern, considered to have a redisplacement risk after closed reduction of > 90% [30].

A majority of patients treated with delayed primary surgery in our study would instead receive early primary surgery under the full implementation of the new guidelines. Not only would a shorter time to surgery reduce the time of immobilization (10 days in our material) and potentially speed up the return to work but it may also benefit the patient in terms of a better final functional outcome. Sirniö et al. concluded that treatment of DRFs with early palmar plating resulted in better 2-year functional outcomes for ≥ 50-year-old patients compared with a primary nonoperative treatment protocol. Delayed surgery in case of secondary displacement was not beneficial in terms of function. [14]. Similarly, Mulders et al. found in a randomized controlled trial that patients treated with early primary surgery had significantly better patient-reported wrist function up to 12 months than patients who received delayed primary surgery [6]. In an earlier study, Abramo et al., a tendency for poorer outcome scores with delayed primary surgery was found in patient-reported outcome [38].

Our study detected a difference between the two study centers in proportions of potential fast-track patients treated with early primary surgery, illustrating that the impact of the new guidelines will vary between hospitals and departments depending on previously used treatment regimens and local traditions. The new treatment recommendations may lead to unnecessary surgery, given that one third of the patients meeting the fast-track criteria were treated nonoperatively in our study. We believe that these include patients with low functional demands, high age, patients declining surgery despite surgeon recommendations, and furthermore fractures with late displacement occurring after radiological follow-up. Future studies are needed to determine the clinical and radiological outcome of these fractures.

Only 5% of the population in our cohort over 65 years of age had low functional demands, defined as domestic care services or institutional living documented in the medical record. The actual number in the regions is about 18% on average for 2014–2017 [39]. Thus, only about one quarter of the expected prevalence was documented. The discrepancy can be explained as this is a retrospective cohort with data collected through patient record review. The incidence of DRFs could even be higher in a more fragile population. For instance, people with dementia have a higher risk of falling, which supports the high incidence of nonoperative treatment in the historical cohort [40]. On the other hand, DRFs in the elderly may be a clinical marker for high physical function. This argument is based on the notion that a patient with low functional demands would be more likely to sustain a hip or vertebral fracture because the simple act of stretching out the arm during a fall is a demonstration of maintenance of function [41]. The new national guidelines may draw attention to surgical treatment of vital elderly patients who may be subject to a conservative regime due solely to their chronological age [7].

Many DRFs with volar comminution were treated nonoperatively. However, evidence suggests that only very few fractures with volar comminution heal in an acceptable alignment. Almost 80% displace on radiological follow-up and of the remaining fractures, another 80% displace later, resulting in a displacement rate of > 95% [35]. Therefore, in patients with volar comminution we do not believe that the new national guidelines will lead to unnecessary surgery.

The retrospective study design has inherent limitations. All clinical results are estimates from medical records and assess only how patients were treated and not how they should have been treated, according to the guidelines. We cannot determine whether patients with nonoperative treatment would have benefited from surgery or vice versa. Moreover, we do not have complete data on suspected osteopenia or osteoporosis. Data have been published showing that patients with DRFs aged 50–75 years have osteopenia or osteoporosis in 83% of cases (75% for men, 84% women) [34], which motivates the use of age > 50 years as a proxy. We believe this is reasonable, with 83% being a strong enough probability of categorizing these patients as having suspected osteopenia or osteoporosis. Lastly, large differences in local treatment traditions exist [12] and therefore the impact of the new national guidelines will vary considerably. However, we present data from two centers close to the national average regarding operative treatment for DRFs according to the Swedish Fracture Registry (Sundsvall: 31.7%, Umeå 28.8%, national average 32.3%). The rates of surgical intervention in Sweden are comparable to those of other countries with a similar standard of living (USA 34% [42], Norway 28% [43]. Based on the Swedish Fracture Registry, the implementation of the guidelines could be further studied on a national level by the Swedish Fracture Registry. Another aspect of evaluating national guidelines is the limited effect that expert criteria may have on surgeons [44].

## Conclusion

The Swedish national guidelines for DRFs may increase the number of early primary surgeries. This increase is estimated to be compensated for by reduced suffering and sick leave, reduced need for return visits, reduced frequency of delayed primary surgeries, reduced frequency of osteotomies and a better functional outcome [30].

Based on the data in the present study, the Swedish national guidelines may risk recommending a few overabundant surgeries. At the same time, a majority of patients treated with delayed primary surgery will be spared 9–13 days in cast waiting for radiological control. When adding to the equation that early primary surgery can benefit the final prognosis, these recommendations seem to be justified.

## Supporting information

S1 Appendix Summary of the Swedish national guidelines. (DOCX)

S1 Data Data for PLOS ONE. All the data used in the study.
(SAV)
