# Supplementary material for: Forecasting effects of “fast-tracks” for surgery in the Swedish national guidelines for distal radius fractures
Source: PLoS One. 2022 Feb 10;17(2):e0260296. doi: 10.1371/journal.pone.0260296 (PMC8830720; doi:10.1371/journal.pone.0260296)
Supplement: S1 Appendix — (DOCX) [file pone.0260296.s001.docx]

**Appendix 1. English translation of the Swedish national guidelines for distal radius fractures.** Full guidelines, as well as an initial summary, can be found in Swedish at <https://d2flujgsl7escs.cloudfront.net/external/Nationellt+v%C3%A5rdprogram+f%C3%B6r+behandling+av+distala+radiusfrakturer.pdf>

= Strong recommendation; assessed as strong after evaluation of pros and cons, scientific evidence, resource consumption as well as the patient’s valuation

= Recommendation; weaker degree of recommendation where the recommendation may change with new knowledge

| Symptoms, clinical findings and diagnostic imaging | The diagnosis distal radius fracture should be made with a conventional x-rays (AP and lateral)  Supplementary CT-scan should in select cases be performed as preoperative investigation  MRI is indicated if there is suspicion of simultaneous carpal fracture |
| --- | --- |
| Non-operative treatment | Treatment differs depending on the patient’s functional demands:  **High functional demands** refer to the need to use the wrist and hand in heavy labor or activities in work, free time or daily activities.  **Intermediate functional demands** is defined as the need to perform activities of daily living (ADL) independently, but without the need to load the wrist heavily in physical labor or spare-time activities.  **Low functional demands** refer to permanent incapability to independently perform activities of daily living (ADLs).  **Patients with high functional demands:** Distal radius fractures should be treated with cast immobilization if the following radiological criteria are met (post reduction):  Dorsal angulation < 10 °  Volar angulation < 15 °  Radial inclination > 15 °  Ulnar variance < 2 mm shortening  Intra-articular step < 2 mm  Volar cortex continuity  Coronal shift < 2 mm  Congruent DRUJ  **Patients with intermediate functional demands:** Distal radius fractures should be treated with cast immobilization if the following radiological criteria are met (post reduction):  Dorsal angulation < 20 °  Volar angulation < 15 °  Radial inclination > 10 °  Ulnar variance < 3 mm shortening  Intra-articular step < 2 mm  Volar cortex continuity  Congruent DRU-joint  **Patients with low functional demands:** Restraint should be exhibited regarding the use of operative treatment for patients with low functional demands. If there is abundant misalignment or compromised distal neurovascular status the fracture should be reduced prior to cast immobilization. Surgery should only be considered if there remains skin, nerve, or circulatory compromise after reduction. |
| Anaesthesia for closed reduction | Local anaesthetic in the fracture hematoma should be used for closed reduction of distal radius fractures. Intravenous regional anaesthesia has benefits compared to hematoma block and can be considered if the competence is available. |
| Reduction technique | When reducing a fracture good knowledge of reduction techniques is needed. |
| Radiological control with  non-operative treatment | Radiological follow-up should be done after approximately 10 days if the patient has **high or intermediate functional demands** if the fracture is dislocated (even within the radiological criteria), if the fracture has been reduced or if the fracture has dorsal comminution. At control the following applies:  If the fracture at control has unchanged position continued cast treatment is recommended.  If the fracture at control has a clearly changed position but still within acceptable radiological criteria surgery should still be considered.  If the fracture at control does not fulfil the radiological parameters in relation to functional demands the patients should be offered surgical treatment.  Radiological control should be avoided if the fracture initially is undisplaced (fissure) and without dorsal comminution.  Radiological control should be avoided if the patient has **low functional demands** and non-operative treatment was planned despite abundant misalignment. |
| Cast immobilization | Cast immobilization for non-operative treatment should end after 4-5 weeks. |
| Operative treatment | Indications for surgery depends on the patient’s functional demands:  **High functional demands** refer to the need to use the wrist and hand in heavy labor or activities in work, free time or daily activities.  **Intermediate functional demands** mean the need to perform activities of daily living (ADL) independently, but without the need to load the wrist heavily in physical labor or spare-time activities.  **Low functional demands** refer to permanent incapability to independently perform activities of daily living (ADLs).  **Patients with high or intermediate functional demands:** should be offered surgical treatment if any of the following criteria are present on initial radiograph, no matter the result of reduction (fast-track):  Volar comminution  Barton fracture (volar or dorsal) with displacement  Smith fracture (volar angulation >15)  Simultaneous presence of dorsal comminution, severe initial displacement (dorsal angulation > 30 °, radial inclination < 10 °, or ulnar variance > 3 mm), and clinical suspicion of physiological osteopenia/osteoporosis.  **Patients with high functional demands**: should be offered surgical treatment if any of the following criteria are present post reduction:  Dorsal angulation > 10 °  Volar angulation > 15 °  Radial inclination < 15 °  Ulnar variance > 2 mm shortening  Intra-articular step > 2 mm  Volar cortex discontinuity  Coronal shift > 2 mm  Incongruent DRU-joint  **Patients with intermediate functional demands**: should be offered surgical treatment if any of the following criteria are present post reduction:  Dorsal angulation > 20 °  Volar angulation > 15 °  Radial inclination < 10 °  Ulnar variance > 3 mm shortening  Intra-articular step > 2 mm  Volar cortex discontinuity  Incongruent DRU-joint  **Patients with low functional demands:** Restraint should be exhibited regarding invasive measures, and surgery should only be considered if there remains skin, nerve, or circulatory compromise after reduction. |
| Choice of surgical method | Dorsally angulated extraarticular or intraarticular undisplaced fracture should be treated with external fixation, percutaneous pinning or volar locking plate.  Fracture with dorsal angulation and intraarticular displacement, comminute volar cortex, Smith fracture or Barton fracture should be treated with volar locking plate. |
| Surgical method; technical recommendations | Volar locking plate:  Modified Henry approach should be used.  The volar locking plate should be placed immediately proximal to the “watershed line” and should lie flush with the volar cortex of the radius.  The distal screws should be placed subchondrally, close to the articular surface.  The screw length of the distal screws should be 2 mm shorter than measured length to the dorsal cortex. At least 4 distal locking screws should be used.  If the plate or screws are placed in a position with increased risk for late complications, a follow up should be planned to evaluate the need of implant removal.  One should strive to repair the pronator quadratus.  External fixation:  Damage to the radial nerve should be avoided when placing the pins.  The pins in the radius and metacarpal bone, respectively, should be parallel in each pair of pins to avoid the risk of fracture between the pins. Predrilling can be helpful.  External fixation should be placed with the wrist fixed straight or in slight extension and without exaggerating the distraction.  External fixation should be controlled with x-ray after approximately 10 days.  Treatment time with external fixation should be 5-6 weeks.  Percutaneous pinning:  Pins can be left under, or outside the skin depending on surgeon’s preference.  Percutaneous pinning should be controlled with x-ray after approximately 10 days. |
| Cast immobilization | After fixation with volar locking plate, cast immobilization for 2 weeks is recommended.  After fixation with pins, cast immobilization for 4-5 weeks is recommended. |
| Simultaneous distal ulnar fracture or DRUJ-instability | In the case of ulnar styloid fracture without simultaneous DRUJ-instability, there is no need for surgical fixation of the ulnar fracture or any change in postoperative plaster treatment.  In case of suspected instability of the DRUJ with or without fracture of the ulnar styloid, intervention should be considered depending on the surgeon’s familiarity with the condition.  There is no support for recommendations regarding concomitant fracture of the distal ulnar metaphysis. |
| Complex fractures and associated soft tissue injuries | Complex radius fractures should be treated by an experienced surgeon in order to minimize the risk for a poor prognosis and complications. |
| Surgical timing | Distal radius fractures that fulfil criteria for surgery should be taken to surgery within one week from the time of the accident.  If surgery is needed after non-operative treatment has been abandoned at 10 days follow-up, surgery should be performed promptly, but with daytime surgery. |
| Perioperative antibiotics | In conjunction with surgery for a closed distal radius fracture 2 g of Cloxacillin should be given intravenously as a single dose. In case of known severe allergy to penicillin, 600 mg of Clindamycin is instead given as a single dose.  In conjunction with surgery for open distal radius fracture 2 g of Cloxacillin should be given intravenously in three doses over the course of a day and then no further prophylactic antibiotic. In case of severely contaminated open fracture, coverage against gram-negative bacteria is advocated with e.g., cefotaxime or piperacillin/tazobactam. In case of severe allergy to penicillin 600 mg of Clindamycin is instead given as three doses over the course of a day. |
| Cast | In emergency care of a distal radius fracture an immobilizing cast in plaster of Paris should be used.  For dorsally angulated fractures the cast splint should be applied dorsally and give three-point support in the form of radial and volar support.  The wrist should be immobilized in functional position (wrist at 30 degrees extension). The cast should allow free movement in the MCP-joints, the CMC 1-joint and the elbow.  Measures to prevent chafing from the cast should be undertaken.  Written and oral cast instructions should be given. |
| Rehabilitation  Early contact and basic rehabilitation measures | All patients, regardless of treatment method and functional demands, should be instructed by healthcare service personnel both orally and in writing regarding movement, edema prophylaxis and activity level.  Contact with physiotherapist or occupational therapist (rehabilitation contact) can be through digital meetings, telephone calls or in-person visit individually or in groups.  Patients who are treated with cast should be offered an initial rehabilitation contact within 1-2 weeks and a second one in conjunction with cast removal.  Patients who are treated surgically should receive an initial rehabilitation contact no later than 3 days after the procedure and a second one in conjunction with cast removal or disassembly of external fixation. |
| Risk factors | Patients who exhibit risk factors for poor prognosis should without delay be offered rehabilitation contact with an in-person visit.  Rehabilitation should be target oriented, individualized and intensified.  There should be clear and established contact paths between physiotherapists/occupational therapist and treating physician. |
| Consult physician | In case of suspicion or detection of abnormal symptoms, feedback should be given to a doctor. |
| Assessment and evaluation after distal radius fracture | Assessment and evaluation of hand and wrist function and activity after distal radius fracture should include both the patient’s own experience and objective parameters. |
